# Variation of Oleanolic and Ursolic Acid in the Flesh of Persimmon Fruit among Different Cultivars

**DOI:** 10.3390/molecules15096580

**Published:** 2010-09-20

**Authors:** Chunhua Zhou, Yanle Sheng, Daqiu Zhao, Zhiqin Wang, Jun Tao

**Affiliations:** 1 College of Horticulture and Plant Protection, Yangzhou University, Yangzhou 225009, China; E-Mails: chzhou@yzu.edu.cn (C.Z.); ylsheng2007@163.com (Y.S.); 405323488@qq.com (D.Z.); 2 College of Agronomy, Yangzhou University, Yangzhou 225009, China; E-Mail: zqwang@yzu.edu.cn (Z.P.)

**Keywords:** persimmon, oleanolic acid, ursolic acid, pentacyclic triterpenoid, HPLC

## Abstract

Oleanolic acid (OA) and ursolic acid (UA) are important bioactive components in many plants, including persimmon (*Diospyros kaki* L.). The present work was carried out to determine OA and UA contents in the flesh of persimmon fruit from 32 cultivars, including 23 astringent and 9 non-astringent ones, by applying high performance liquid chromatography (HPLC) with UV detection. Both OA and UA were present in all of the investigated cultivars, except for three, ‘Hiratanenashi’, ‘Ribenhongshi’ and ‘Matsumotowase’. The OA content ranged from traces to 88.57 μg/g FW, and that of UA were between traces and 27.64 μg/g FW.

## 1. Introduction

Persimmon (*Diospyros kaki* L., Ebenaceae) is a major horticultural crop of China, and is widely cultivated in other parts of Northeast Asia, including Korea, and Japan [1]. Persimmon fruits are rich in different kinds of nutrients and phytochemicals such as carbohydrates, organic acid, vitamins, tannins, polyphenols, dietary fiber, triterpenoids, and carotenoids, which contribute significantly to their taste, colour, nutritive and medicinal values [2,3,4,5,6,7,8,9,10]. 

**Figure 1:**
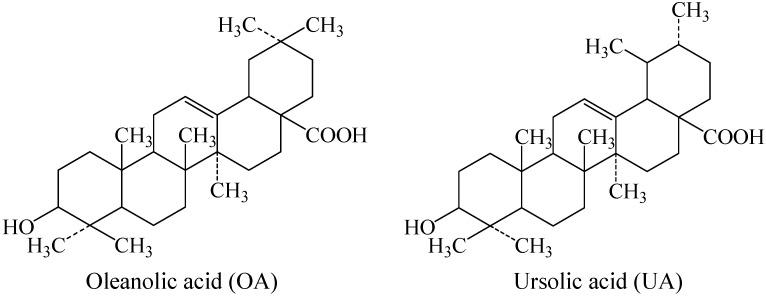
Chemical structures of OA and UA.

The isomeric pentacyclic compounds oleanolic acid (OA) and ursolic acid (UA) (Figure 1) are two common triterpenoids found in *D. kaki* leaves [1,11]. Both compounds have bioactivities such as anti-inflammatory [12], hepatoprotective [13], gastroprotective [14], cardiovascular [15], anti-tumor [16], anti-HIV [17] and immunoregulatory effects [18]. Zheng *et al.* [10] detected triterpenoids such as OA and UA from the pedicle, Ma *et al.* [7] also found these two components in persimmon frost (the white substance on dried persimmon). Liu and Xie [6] isolated five ursolate compounds from persimmon fruit by HPLC and identified them by means of spectral analysis (IR, MS, ^1^H-NMR, ^13^C-NMR). The objective of this work was to identify and quantify OA and UA in the flesh of persimmon fruit by HPLC, for a better nutritive evaluation and medical utilization of persimmon fruits in the future.

## 2. Results and Discussion

### 2.1. Evaluation of Fruit Quality

The fruit size, shape, TSS (Total Soluble Solid) content and titratable acid content varied due to the different persimmon cultivars, no matter whether astringent or non-astrigent (Table 1). The differences in fruit shapes among all the astringent persimmon cultivars was larger than that among the non-astrigent persimmon cultivars, and the average weight of individual fruit of the astringent persimmon cultivars was heavier than that of the non-astrigent persimmons. The average TSS content of the astringent persimmon cultivars was lower than that of the non-astrigent persimmons, however, the titratable acid was on the reverse trend. The great differences of quality indexes among different cultivars might result from genetic and geographical origins.

**Table 1:** Main Fruit Quality Index of Persimmon Cultivars.

Cultivars	W *^a^* (g)	VD *^b^* (cm)	HD *^c^* (cm)	FSI *^d^*	TSS *^e^* (ºBrix)	TA *^f^* (%)
*Astringent persimmons*						
Boaidashuishi	53.29	3.34	4.94	0.68	13.75	0.22
Heixinshi	94.73	5.05	5.61	0.90	11.11	0.37
Heshi	339.35	6.63	9.38	0.71	16.25	0.19
Hiratanenashi	113.94	4.18	6.29	0.66	15.64	0.19
Hiro	101.73	4.82	5.92	0.81	15.20	0.18
Jianshi	122.70	6.33	5.69	1.11	16.31	0.30
Jinchengxiaoshi	66.27	3.67	5.22	0.70	16.80	0.18
Jinshi	277.98	7.56	7.45	1.01	13.24	0.20
Lantianshuishi	148.58	5.69	6.48	0.88	14.26	0.26
Mantianhong	209.03	5.92	7.72	0.77	16.57	0.17
Mendunshi	81.66	5.05	5.45	0.93	18.15	0.12
Miandanshi	87.20	4.92	5.37	0.92	17.60	0.33
Mimiguan	48.28	4.41	4.33	1.02	15.48	0.38
Naiyoushi	141.28	5.17	6.47	0.80	13.45	0.24
Ribenhongshi	96.64	4.29	5.82	0.74	18.57	0.17
Shagu NO.1	106.00	4.72	5.97	0.79	15.01	0.23
Tianfushi	123.34	4.96	6.22	0.80	14.40	0.14
Tonewase	215.10	5.57	7.95	0.70	17.50	0.20
Xiaoercao	35.72	3.19	4.30	0.74	12.38	0.24
Xingyangbaheshi	84.16	4.09	5.58	0.73	14.94	0.153
Yichuanling	63.28	4.42	4.72	0.94	15.20	0.14
Yueshi	153.82	4.69	5.79	0.81	14.90	0.27
Zhaotianhong	159.40	4.86	7.21	0.67	15.508	0.20
Average	127.11	4.94	6.08	0.82	15.31	0.22
*Non-astringent persimmons*						
Hanagosho	111.90	5.16	6.39	0.81	21.58	0.09
Jiro	114.54	4.41	6.64	0.66	13.34	0.11
Luotiantianshi	57.02	4.01	4.78	0.84	15.65	0.14
Matsumotowase	59.20	4.21	5.21	0.81	17.19	0.15
Nishimurawase	115.58	4.57	6.75	0.68	16.39	0.21
Okugosho	100.68	4.56	6.16	0.74	18.16	0.27
Suruga	93.38	4.75	6.08	0.78	13.19	0.09
Xianxitianshi	110.40	4.56	6.49	0.70	15.33	0.14
Zenjimaru	135.65	5.29	6.43	0.82	14.10	0.21
Average	99.81	4.61	6.10	0.76	16.10	0.16

*^a^* W: Weight; *^b^* VD: Vertical diameter; *^c^* HD: Horizontal diameter; *^d^* FSI: Fruit shape index, The ratio of VD/HD; *^e^* TSS: Total soluble solid; *^f^* TA: titrable acid.

### 2.2. Identification of OA and UA in the Flesh of Persimmon Fruits by HPLC

**Figure 2:**
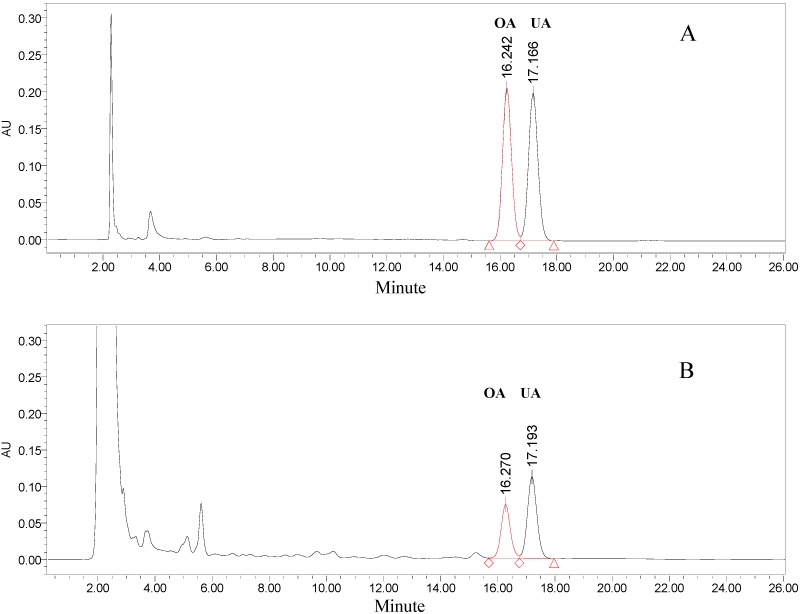
HPLC chromatograms of OA, UA standard (A) and the flesh extract of persimmon fruit (B).

OA and UA standards were detectable under a wavelength of 210 nm. At this wavelength, OA and UA could be eluted efficiently and simultaneously detected by a mobile phase consisted of methanol (A) and 0.03 mol/L phosphate buffer (pH 2.8) (B) with a ratio of 88:12 (A:B, v/v). With a flow rate of 1 mL/min, the chromatographic retention times of OA and UA were about 16.24 and 17.17 min, respectively (Figure 2A). This HPLC system successfully separated and simultaneously identified the OA and UA in the flesh of persimmon fruit (Figure 2B), and can be used to quantify the content of OA and UA.

### 2.3. OA and UA Contents in the Flesh of Persimmon Fruit

Based on the above detection method, the OA and UA content in the flesh of persimmon fruits from 32 cultivars, including 23 astringent and 9 non-astringent ones were analyzed, and the results are shown in Table 2. Both OA and UA were detected in the flesh of all cultivars except for ‘Hiratanenashi’, ‘Ribenhongshi’ and ‘Matsumotowase’. In the flesh of ‘Hiratanenashi’, OA was under the detection limit, while in ‘Ribenhongshi’ and ‘Matsumotowase’, UA was undetectable. The highest OA content was detected in ‘Ribenhongshi’, and the highest UA content was detected in ‘Jinchengxiaoshi’. The contents of OA and UA in the fleshes of different persimmon cultivars were significantly different. The contents of OA and UA were trace ~ 88.57 μg/g FW and trace ~ 27.64 μg/g FW for astringent cultivars, respectively, while the contents of OA and UA were 1.24 ~ 26.43 μg/g FW and trace ~ 12.23 μg/g FW for non-astringent cultivars, respectively. The results of Cui *et al.* [19] and Guo *et al.* [20] also demonstrated that the OA and UA contents of *Ziziphus jujuba* and *Crataegus pinnatifida* fruits varied great with the cultivars. However, whether genetic or enviromental factors cause such difference deserve further study. 

**Table 2:** OA and UA Content in the Flesh of Persimmon Fruits (μg/g FW).

Cultivar	OA	UA
*Astringent persimmons*		
Boaidashuishi	4.39 ± 0.05	11.18 ± 0.00
Heixinshi	20.44 ± 0.59	2.23 ± 0.15
Heshi	4.72 ± 0.29	12.88 ± 0.96
Hiratanenashi	*	1.44 ± 0.02
Hiro	1.89 ± 0.33	18.87 ± 1.03
Jianshi	3.87 ± 0.23	11.08 ± 0.30
Jinchengxiaoshi	1.70 ± 0.00	27.64 ± 0.01
Jinshi	0.57 ± 0.01	0.30 ± 0.00
Lantianshuishi	1.09 ± 0.04	16.83 ± 0.81
Mantianhong	5.40 ± 0.00	17.61 ± 0.20
Mendunshi	19.62 ± 1.51	0.23 ± 0.01
Miandanshi	4.88 ± 0.18	15.30 ± 0.24
Mimiguan	0.59 ± 0.03	3.74 ± 0.09
Naiyoushi	0.16 ± 0.01	1.06 ± 0.04
Ribenhongshi	88.57 ± 1.91	*
Shagu NO.1	4.28 ± 0.99	14.89 ± 1.13
Tianfushi	1.57 ± 0.01	3.03 ± 0.02
Tonewase	46.42 ± 0.32	1.79 ± 0.02
Xiaoercao	18.94 ± 0.38	2.12 ± 0.09
Xingyangbaheshi	1.16 ± 0.12	2.00 ± 0.03
Yichuanling	5.60 ± 0.33	14.67 ± 0.57
Yueshi	1.15 ± 0.01	2.82 ± 0.05
Zhaotianhong	0.74 ± 0.10	22.74 ± 0.04
Average	10.81	9.29
*Non-astringent persimmons*		
Hanagosho	12.33 ± 0.43	2.23 ± 0.03
Jiro	26.43 ± 0.24	0.24 ± 0.03
Luotiantianshi	22.90 ± 0.20	7.09 ± 0.02
Matsumotowase	20.85 ± 0.14	*
Nishimurawase	12.80 ± 0.12	0.24 ± 0.01
Okugosho	1.31 ± 0.06	1.47 ± 0.02
Suruga	1.24 ± 0.01	1.81 ± 0.03
Xianxitianshi	15.37 ± 0.57	1.03 ± 0.33
Zenjimaru	5.68 ± 0.55	12.23 ± 1.18
Average	13.21	3.29

* in trace amounts.

## 3. Experimental

### 3.1. Materials and Reagents

Persimmon fruits of 32 cultivars were used in this study, including 23 astringent and 9 non-astringent ones (Table 1). During the persimmon edible period, the fruits of different persimmon cultivars were collected from National Germplasm Nursery of Persimmon (Yangling, Shanxi, China), Zhenjiang Agricultural Science Institute (Jurong, Jiangsu, China) and Persimmon Germplasm Experiment Station of Huazhong Agricultural University (Wuhan, Hubei, China) and then transferred to the lab. Ten intact fruits in each cultivar were selected to determine the main quality indexes, including the fruit size, shape, TSS content and titratable acid content, and the results were listed in Table 1. After that, the pedicle and peel were removed, the flesh was cut into small pieces and frozen in liquid nitrogen, and then stored at -20 °C for analysis. OA (TCM-031, purity≥98%) and UA (TCM-036, purity≥98%) standards were purchased from Nanjing TCM Institute of Chinese Materia Medica (Nanjing, Jiangsu, China). Methanol (HPLC grade) was obtained from Caledon Laboratories Co. (Georgetown, Ont., Canada). All the other reagents used in the present study were of analytical grade.

### 3.2. Preparation of Standard Solution and Crude Extract

A stock solution of 1 mg/mL was prepared in methanol for OA and UA standards, respectively. A serial dilution was made on each stock solution with methanol to prepare standard solutions at concentrations of 50, 100, 200, 300, 400 μg/mL, from each of which 20 μL was used for plotting the standard curves for OA and UA, respectively. For preparation of crude extract, suitable amount of flesh material was collected and fully grinded with liquid nitrogen. Subsequently, sample (2 g) was weighed and transferred to a 25 mL centrifuge tube, and ethanol (10 mL) was added in and mixed homogeneously, and then extracted for 30 min by KQ-500B ultrasonic cleaning machine (Kunshan Ultrasonic Equipment Co. Ltd., Kunshan, Jiangsu, China). The sample was centrifugated at 8,000 g for 10 min, and then the filtrate was collected. Precipitates were extracted again and both filtrates were pooled and evaporated to dryness at 35℃. The residue was dissolved in 1 mL methanol and transferred to an Eppendorf tube. The crude extract was filtered though a 0.22 μm micro-filter before HPLC analysis.

### 3.3. HPLC Analysis of OA and UA

Quantifications of OA and UA were performed on a Waters-2695 HPLC system (Beckman Coulter, USA) equipped with Waters 2695 pump, 2487 UV detector. The column was a Lichrospher C_18_ (4.6 × 250 mm, 5µm) (Hanbon Sci. and Tech, China) equipped with a guard column with the same stationary phase. All these two compounds were detected at 210 nm at room temperature with an eluent flow rate of 1.0 mL/min and an injection volume of 20μL, as previously described [21]. The mobile phase consisted of methanol (A) and 0.03 mol/L phosphate buffer (pH 2.8) (B) with a ratio of 88:12 (A:B, v/v) for simultaneous detection of OA and UA. 

### 3.4. Statistical Analysis

All data are means of three replicates with standard deviations. Microsoft Excel (Microcal Software Inc., Northampton, MA, USA) was used to calculate standard deviations.

## 4. Conclusions

In this study, chromatograms of OA and UA in the flesh of persimmon fruit were well established, and the content of OA and UA in the flesh of different cultivars of astringent and non-astringent persimmons were analyzed. The results suggested that both OA and UA were present in almost of all the investigated cultivars, except for ‘Hiratanenashi’, ‘Ribenhongshi’ and ‘Matsumotowase’. The OA content ranged from traces to 88.57 μg/g FW, and that of UA were between traces and 27.64 μg/g FW. These results may provide a theoretical foundation for the medical utilization of persimmon fruits in the future.
